# Alkaline stress reduces root waving by regulating PIN7 vacuolar transport

**DOI:** 10.3389/fpls.2022.1049144

**Published:** 2022-12-13

**Authors:** Yu Liu, Chenglin Mu, Dongdong Du, Yi Yang, Lixin Li, Wei Xuan, Stefan Kircher, Klaus Palme, Xugang Li, Ruixi Li

**Affiliations:** ^1^ State Key Laboratory of Crop Biology, College of Life Sciences, Shandong Agricultural University, Tai’an, China; ^2^ Key Laboratory of Molecular Design for Plant Cell Factory of Guangdong Higher Education Institutes, Institute of Plant and Food Science, School of Life Sciences, Southern University of Science and Technology, Shenzhen, China; ^3^ Sino-German Joint Research Center on Agricultural Biology, College of Life Sciences, Shandong Agricultural University, Tai’an, China; ^4^ Key Laboratory of Saline-alkali Vegetation Ecology Restoration, Ministry of Education, College of Life Sciences, Northeast Forestry University, Harbin, China; ^5^ State Key Laboratory of Crop Genetics and Germplasm Enhancement and MOA Key Laboratory of Plant Nutrition and Fertilization in Lower‐Middle Reaches of the Yangtze River, Nanjing Agricultural University, Nanjing, China; ^6^ Institute of Biology II/Molecular Plant Physiology, Faculty of Biology, Albert-Ludwigs-University of Freiburg, Schänzlestr. 1, Freiburg, Germany

**Keywords:** alkaline stress, root waving, PIN7, FREE1, PAT

## Abstract

Root development and plasticity are assessed *via* diverse endogenous and environmental cues, including phytohormones, nutrition, and stress. In this study, we observed that roots in model plant *Arabidopsis thaliana* exhibited waving and oscillating phenotypes under normal conditions but lost this pattern when subjected to alkaline stress. We later showed that alkaline treatment disturbed the auxin gradient in roots and increased auxin signal in columella cells. We further demonstrated that the auxin efflux transporter PIN-FORMED 7 (PIN7) but not PIN3 was translocated to vacuole lumen under alkaline stress. This process is essential for root response to alkaline stress because the *pin7* knockout mutants retained the root waving phenotype. Moreover, we provided evidence that the PIN7 vacuolar transport might not depend on the ARF-GEFs but required the proper function of an ESCRT subunit known as FYVE domain protein required for endosomal sorting 1 (FREE1). Induced silencing of *FREE1* disrupted the vacuolar transport of PIN7 and reduced sensitivity to alkaline stress, further highlighting the importance of this cellular process. In conclusion, our work reveals a new role of PIN7 in regulating root morphology under alkaline stress.

## Introduction

Alkaline stress is severe environmental stress affecting the growth of plants, particularly those growing in saline–alkaline soil. High alkaline pH is known to threaten plant growth *via* misbalancing acid–base, ions, or molecules by affecting plant metabolism (Shi and Sheng, 2005; Cha-Um et al., 2009; Patil et al., 2012; Li et al., 2015). It is estimated that approximately 954 million hectares of saline–alkaline soil is present worldwide (Ayyub et al., 2015). Therefore, elucidating the mechanisms underlying plant responses to alkaline stress will establish the foundation for expanding agricultural land and increasing crop yield in the future.

Roots are essential organs for plant anchoring, nutrition, and water absorption. Roots exhibit a high degree of adaptive plasticity in response to different endogenous and environmental factors (Overvoorde et al., 2010; Petricka et al., 2012; Lavenus et al., 2013; Laskowski, 2013; Motte et al., 2019). In a classic study, the roots of plants grown on the surface of an inclined agar plate showed a pattern characterized by alternating left and right movements during growth in constant gravity, thus exhibiting a rhythmically curved root waving phenotype (Yuen et al., 2005; Mochizuki et al., 2005; Sakai et al., 2012). Root rhythmic waving is particularly important during growth to prevent obstacles encountered in the soil. Moreover, root is the major plant organ to sense soil alkalinity and therefore responds to alkaline stress. It has been shown that alkaline conditions inhibit primary root elongation by reducing cell division in the root apical meristem (RAM). During this process, alkaline stress may affect the auxin influx transporter AUXIN1 (AUX1) and stimulate the expression of auxin biosynthesis-related genes *via* the ethylene signaling pathway, ultimately leading to increased auxin content and the inhibition of root elongation (Li et al., 2015). The auxin efflux transporter PIN-FORMED 2 (PIN2) is reported to be involved in root adaptation to alkaline stress *via* regulating proton secretion (Xu et al., 2012). In addition, proton secretion mediated by the plasma membrane (PM) H^+^-ATP synthase (H^+^-ATPase) has been shown to play an essential role in plant response to alkaline stress (Fuglsang et al., 2007; Yang et al., 2010; Xu et al., 2012). The protein kinase SOS2-LIKE5 (PKS5) inhibits the activity of PM H^+^-ATPase by preventing its interaction with the adapter 14-3-3 proteins. A previous study showed that the *pks5* mutant exhibits greater alkaline stress tolerance than wild-type (WT) plants (Fuglsang et al., 2007; Xu et al., 2012). Another study reported that the chaperone J3-deficient mutant *j3* is hypersensitive to alkaline stress due to enhanced H^+^-ATPase activity (Yang et al., 2010). Further, the same group found that the protein SCaBP3 (SOS3-LIKE CALCIUM BINDING PROTEIN 3)/CBL7(CALCINEURIN B-LIKE 7) senses Ca^2+^ signals, after which the protein is dissociated from AHA2 to promote PM H^+^-ATPase activity. This leads to the development of resistance to alkaline stress in the *scabp3* mutant (Yang et al., 2019).

Auxin plays a key role in root growth and root adaptation to endogenous and environmental cues, which include root apical meristem maintenance, root orientation, root elongation, lateral root formation, and root gravitropism (Overvoorde et al., 2010; Strohm et al., 2012; Tian et al., 2013; Ogura et al., 2019; Kleine-Vehn et al., 2010). Moreover, it is involved in crosstalk with other hormones, such as cytokinin, ethylene, jasmonic acid, abscisic acid, and brassinosteroid to fine-tune root growth (Buer et al., 2003; Aloni et al., 2004; Li et al., 2005; Han et al., 2009; Staswick, 2009). The major function of auxin is to regulate cell-specific transcription of target genes *via* the auxin signaling pathway. This pathway comprises three major protein families: TIR1/AFB F-box auxin receptors (Dharmasiri et al., 2005a; Dharmasiri et al., 2005b; Mockaitis and Estelle, 2008), Aux/IAA transcriptional repressors (Remington et al., 2004; Overvoorde et al., 2005), and auxin response factor (ARF) transcription factors (Okushima et al., 2005; Guilfoyle and Hagen, 2007).

Auxin is usually synthesized in young plant organs (e.g., shoots) and is then transported to target organs *via* polar auxin transport (PAT). This transport relies on auxin polar transporters, such as AUX1 and AUX1-LIKEs (LAXs) (Blakeslee et al., 2019), PINs, and several ATP-binding cassette B (ABCB)/P-glycoprotein (PGP) proteins (Friml et al., 2002; Titapiwatanakun and Murphy, 2009; Grunewald and Friml, 2010; Qu et al., 2017). PINs exhibit polar localization at the PM, which defines the direction of auxin flow and generates an auxin gradient in the roots (Benkova et al., 2003; Blilou et al., 2005; Adamowski and Friml, 2015). The polar localization of PIN1 and PIN2 is maintained by constitutive endocytosis and recycling (Pan et al., 2009). The ARF-guanine nucleotide exchange factor (GEF) GNOM and retromer component SORTING NEXIN1 (SNX1) are reported to regulate the endomembrane sorting of PIN proteins with an essential role in plant growth and development (Geldner et al., 2003; Jaillais et al., 2006). GNOM plays an important role in the recycling of PIN protein from endosomes back to PM (Steinmann et al., 1999; Geldner et al., 2003; Jaillais and Gaude, 2007). SNX proteins participate in the sorting of specific PM transporters on the trans-Golgi network/early endosomes (TGN/EE) (Heucken and Ivanov, 2018). Ubiquitinated PIN2 can also be sorted into vacuoles for degradation. A unique plant endosomal sorting complex required for transport (ESCRT) protein FYVE domain protein required for endosomal sorting 1 (FREE1) is reported to regulate PIN2 vacuolar transport (Gao et al., 2014). FREE1 is essential for the biogenesis of multivesicular bodies (MVBs) and other intracellular transport pathways, including vacuolar protein transport and autophagy pathway. *free1* knockout plants fail to form the intraluminal vesicles (ILVs) in MVBs, impair vacuolar transport and accumulate a large number of autophagosomes outside the vacuole (Raiborg and Stenmark, 2009; Henne et al., 2011; Gao et al., 2014; Gao et al., 2015; Zhao et al., 2015). Therefore, vesicle transport components are actively involved in regulating the dynamics of PIN proteins.

Here, we report new findings that help elucidate the role of PIN7 in regulating root responses to alkaline stress. Under normal (pH 5.8) growth conditions, roots of wild-type *Arabidopsis* plants exhibit waving and oscillating growth patterns, which attenuate and grow in a straight line instead under alkaline growth conditions (pH 8.0). We next demonstrated that PIN7 exhibits obvious intracellular translocation into vacuole lumen in response to alkaline stress. This process is mediated *via* the FREE1-mediated vacuolar sorting pathway. *free1* knock-down mutants disrupt the vacuolar transport of PIN7 and reduce sensitivity to alkaline stress, highlighting the importance of this cellular process. Based on the data reported in this study, we propose that PIN7 is a key factor in root adaptation to alkaline stress.

## Materials and methods

### Plant material

The plant materials used in this work were *Arabidopsis thaliana* ecotype Columbia-0 (Col-0), Landsberg *erecta* (L*er*), *PIN3_pro_:PIN3–GFP* (Blilou et al., 2005), *PIN7_pro_:PIN7–GFP* (Blilou et al., 2005), *nph4* (Stowe-Evans et al., 1998), *tir1-1* (Dharmasiri et al., 2005a), *msg2-1* (Tatematsu et al., 2004), *mCherry- VAMP711* (Geldner et al., 2009), *HAP13-RFP* (Wang et al., 2013 *)*, *FREE1 DEX-RNAi* (Gao et al., 2014), *eir1-1* (Luschnig et al., 1998), T-DNA mutants *pin3-4* (Young et al., 2006), and *snx1-2* (Zhang et al., 2022), transposon insertion mutant *pin7* (GT4114; Martienssen, 1998). For plant propagation, seeds were first surface sterilized with 75% ethanol for 5 min, followed by incubation with 2.6% NaClO for 10 min before being rinsed five times with water.

### Growth condition

Seeds were grown on 1/2 Murashige and Skoog (MS) medium (HB8469; Hai bo, China) with concentrations of 0.8%, 1%, 1.3%, or 1.5% sugar, and 1.8% agar (A8190; Solarbio, China). Sowing plates were maintained at 4°C for 2 days before transferred to a light incubator. Seeds were germinated at 75 degrees tilted solid medium in an illumination incubator (Ningbo Ledian equipment manufacturing, China) equipped with uniform LED light, 16 h light/8h dark cycle at 22°C.

### Chemical treatment

**Figure 1:**
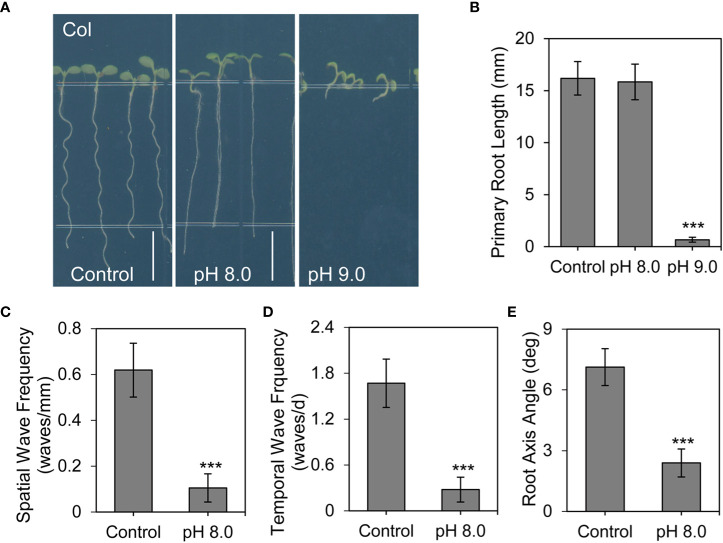
Root waving is inhibited under alkaline stress conditions. **(A)** Root phenotype of wild-type Arabidopsis seedlings on growth medium at pH 5.8 (control), pH 8.0, or pH 9.0 for 6 days (n > 35). Scale bar, 5 mm. **(B)** Quantification of the primary root length as shown in **(A)** Values are mean ± SD (n > 35 seedlings). **(C, D, E)** Statistical analysis of the spatial wave frequency (waves/mm) **(C)**, temporal wave frequency (waves/d) **(D)**, and root axis angle **(E)** as shown in **(A)** Each experiment was repeated three times (n > 35). Statistically significant differences were determined by using student’s t-test (***P < 0.001).

Dexamethasone (DEX) dissolved in ethanol was used (1:1000) to induce *FREE1* gene silencing. For short term treatment, *Arabidopsis* seeding were treated with 5 μM DEX in 1/2 MS liquid medium for 1 d (**Figure S5**). For long term treatment, seedlings were germinated on 1/2 MS solid medium containing 10 nM DEX for 6 d before recording (**Figure 1**).

For Brefeldin A (BFA) or wortmannin treatment, seedlings were grown on 1/2 MS medium for 5 days and then transferred to 1/2 MS medium containing 50 μM BFA or 33 μM wortmannin for overnight incubation. Both chemicals were dissolved in dimethyl sulfoxide (DMSO) for stock solution and diluted to working solution in 1:1000 ratio.

For the KCl experiment, KCl at final concentrations of 1.3 mM and 2.2 mM (according to the concentrations of potassium ion (K^+^) in media at pH 8.0 and 8.5, respectively) was added to 1/2 MS medium.

### Confocal microscopy

All fluorescence images were visualized using a Zeiss LSM 510 Meta microscope (Carl Zeiss Microimaging). For Green Fluorescent Protein (GFP) imaging, we used an excitation wavelength of 488 nm and emission wavelength of 505–530 nm. For Propidium Iodide (PI) and Red Fluorescent Protein (RFP) imaging, we used an excitation wavelength of 561 nm and an emission wavelength of 590–635 nm. A Zen 2016 (Carl Zeiss Microimaging) microscope system was used to process the images.

### Western blotting

Proteins were extracted from 7-day-old seedlings using an extraction buffer containing 50 mM Tris–HCl (pH 7.5), 10 mM EDTA, 2 mM EGTA, 0.1% SDS, 1 mM DTT mixture, and 0.1 mM PMSF. After centrifugation at 12,000 rpm and 4°C for 10 min, the supernatant containing the total protein was collected. For Western blotting, we first denatured proteins at 96°C in 5× SDS-PAGE loading buffer (1:4). These proteins were run on a 12% SDS-PAGE gel, and then was transferred to a nitrocellulose membrane *via* blotting. The blot was probed with mouse anti-GFP monoclonal antibody (1:1200), and the GFP signal was detected using an HRP-conjugated anti-mouse antibody (1:1000) (Beyotime Biotechnology). Intracellular TUB3 was used as a loading control (Qi et al., 2020). Protein molecular weight markers (20350ES72; YEASEN, China) were used as size standards in gel electrophoresis. The membranes were visualized using an enhanced chemiluminescence substrate and a Tanon 5200 multi-imaging system (Shanghai, China).

### Statistical analyses

In this study, we repeated all experiments at least three times to obtain replicates. For each experiment, we used at least 30 seedlings in the alkaline treatment and control groups. We used Student’s t-test to analyze the statistical significance. All data were presented as mean ± standard deviation.

Root length and root axis angle were measured using ImageJ. The primary root length was measured from the junction between root and hypocotyl to root tip. The root spatial wave frequency was recorded as the root wave number per millimeter. The temporal wave frequency was recorded as the root wave number per day. The root axis angle was measured by the offset angle of the root growth direction to gravity.

**Figure 2:**
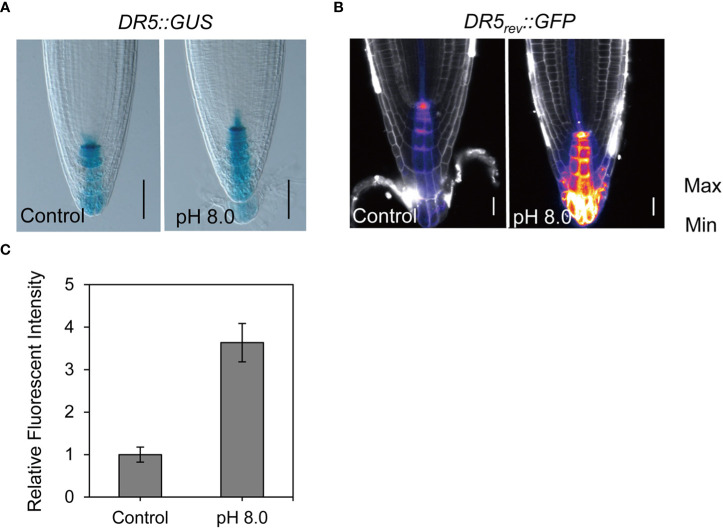
Alkaline stress increases the auxin signal in root. **(A)** Staining renderings of the auxin reporter *DR5::GUS*. The *DR5::GUS* transgenic seedlings were grown at pH 5.8 (control) or pH 8.0 for 6 d before recording. Scale bar, 50 μm. Increased GUS signal indicates elevated auxin level. **(B)** False color of *DR5rev::GFP* images helps to visualize the fluorescence intensity. **(C)** Statistical analysis of DR5rev::GFP reporter as shown in **(B)**. Auxin reporter lines *DR5rev::GFP* were grown at pH 5.8 (control) or pH 8.0 for 6 d before imaging. n > 20. Scale bar, 20 μm. Statistically significant differences were determined by using student’s t-test (*** P < 0.001).

**Figure 3:**
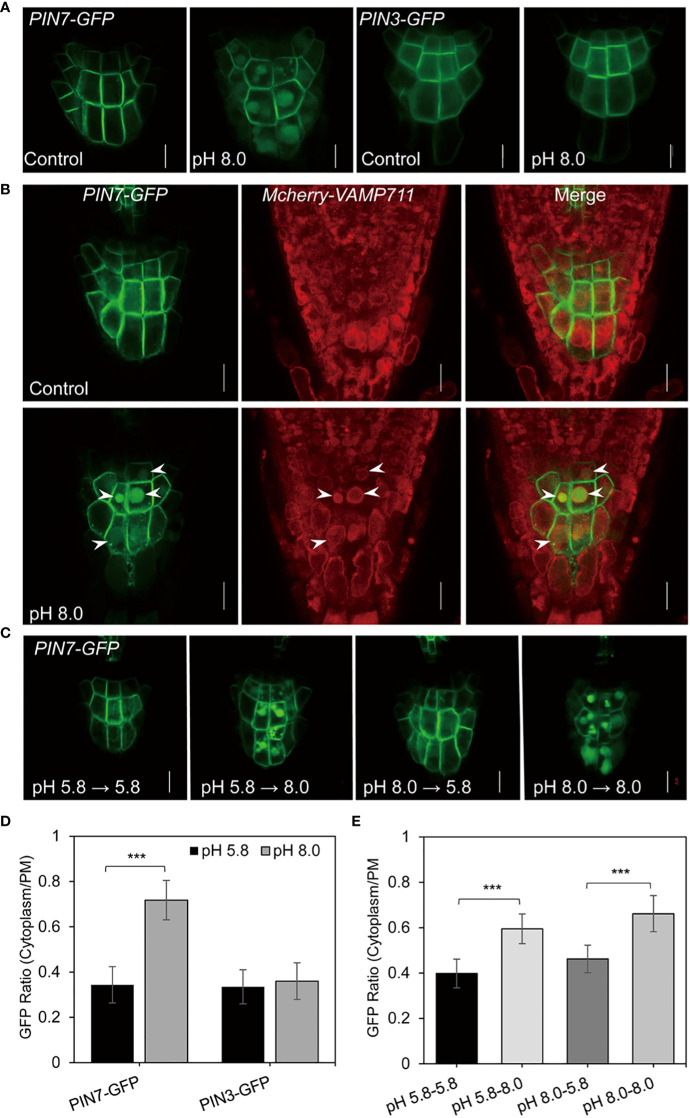
PIN7 is transported to the vacuole lumen under alkaline stress. **(A)** The subcellular localization of PIN3 and PIN7 in columella cells of 6-d-old seedlings under control (pH 5.8) or alkaline (pH 8.0) conditions, respectively. Scale bar, 10 mm. **(B)** PIN7 is transported to vacuole lumen under alkaline conditions. A crossed line between PIN7–GFP and the tonoplast marker mCherry-VAMP711 was grown under control (pH 5.8) or alkaline (pH 8.0) conditions for 6 days (n > 10) before imaging. The arrows indicate the co-localized signal between PIN7–GFP and mCherry-VAMP711- labeled tonoplast. Scale bar, 10 mm. **(C)** The change of subcellular localization of PIN7 under alkaline conditions is reversible. PIN7–GFP seedlings were grown under control (pH 5.8) or alkaline (pH 8.0) conditions for 3 days and then transferred to pH 5.8 or pH 8.0 medium plates for another 3 days before imaging. Scale bar, 10 mm. **(D, E)** Quantitative analysis of the signal ratio between cytoplasm and plasma membrane (PM) in columella cells of PIN7-GFP **(D)** and PIN3-GFP **(E)** transgenic seedlings. Statistically significant differences were determined by using student’s t-test (***P < 0.001).

The false color of *DR5rev::GFP* images was generated by using the split channels of ImageJ. The fluorescence intensity was visualized by adding color from image lookup tables (**Figure 2B**). The signal intensity of *DR5rev::GFP* was quantified by polygon selections to calculate integrated density with ImageJ (**Figures 2C**, **3G**; **Figure S2D**).

The signal intensity of cytoplasmic PIN7-GFP and PIN3-GFP were measured by using polygon selections to calculate the mean of integrated density with ImageJ. The signal intensity of PM-localized PIN7 and PIN3 were measured by using the segmented line to calculate the mean of integrated density with ImageJ.

## Results

### High pH affects root morphogenesis

To investigate how plants respond to alkaline environment, we examined root development under different pH conditions. Consistent with the results from previous studies (Mochizuki et al., 2005; Yuen et al., 2005; Sakai et al., 2012), roots of Col seedlings exhibited a periodic wave and skewed growth pattern under control conditions (pH 5.8 on 1/2 MS medium). Root development was severely attenuated at very high pH alkaline conditions (pH 9.0) (**Figures 1A, B**), preventing further analysis of root phenotypes. However, under moderate high pH alkaline conditions (pH 8.0), the primary root growth was not obvious changed but the root waving pattern was significantly inhibited (**Figures 1A, B**). Furthermore, statistical analyses indicated that alkaline conditions at pH 8.0 also reduced the spatial and temporal wave frequency (**Figures 1C, D**). Roots of wild-type plants usually skew rightward under control conditions (Buer et al., 2003). However, at pH 8.0, we found that the root axis angle was significantly smaller than that under control conditions (**Figure 1E**). Thus, alkaline stress disturbed the root waving pattern.

Next, we examined other possible causes of these phenotypes by assessing the effect of K^+^ and agar concentration in the growth medium. Under high pH conditions, the agar is harder than that under normal (pH 5.8) conditions. Despite this additional hardness, our results showed that the root length and root waving phenotype of wild-type seedlings were similar in plates with different concentrations of agar (**Figures S1A** **–E**). In addition, we attempted to exclude the effect of K^+^ concentration on the root waving phenotype because KOH was used to adjust the pH of the medium. Our results showed that the root waving phenotype was also similar in medium with different concentrations of K^+^ (**Figures S1F** **–J**).

Altogether, these results suggest that the defects in root waving phenotype were mainly associated with alkaline stress. Based on the above results, we used pH 8.0 as the alkaline stress condition and pH 5.8 as the control condition for further analysis of the root waving phenotype.

### Auxin is involved in the regulation of root waving

It is well known that auxin plays a crucial role in the establishment of root architecture. Therefore, we first evaluated the auxin response to alkaline conditions using the auxin markers *DR5rev::GFP* and *DR5::GUS* (Sabatini et al., 1999). Under control conditions, the auxin maxima were observed in the quiescent center. However, under high pH conditions, the expression of the *DR5::GUS* and *DR5rev::GFP* signal was increased, particularly in the columella cells of root cap (**Figures 2A, B**). This suggests that alkaline stress leads to an increase of auxin level. To test this hypothesis, we further assessed *DII-VENUS*, a negative auxin response marker (Brunoud et al., 2012). As expected, the VENUS signal was decreased dramatically in the epidermis, cortex, and stele regions at pH 8.0 (**Figure S2A**), confirming that the auxin signal indeed increased in meristem cells. In addition, the signal intensity of columella marker *PET111* was increased at pH 8.0, indicating that the identity of columella cells was altered in response to alkaline stress (**Figure S2B**). To explore whether the alkaline response was associated with the auxin signaling pathway, we evaluated the *DR5rev::GFP* response to alkaline stress in different auxin signaling mutants, including *tir1-1* (Dharmasiri et al., 2005a), *msg2-1* (Tatematsu et al., 2004), and *nph4* (Stowe-Evans et al., 1998). Our data showed that the GFP signals also increased in these mutants at pH 8.0 similar to what we have observed in the wild-type control seedlings (**Figures S2C, D**), thereby indicating that the altered auxin response under alkaline stress may be independent of the auxin signaling pathway.

### High pH conditions alter the subcellular localization of PIN7

The auxin gradient depends on PIN-mediated PAT. Previous studies reveal that the membrane localization of PIN proteins is essential for efficient PAT (Friml and Palme, 2002; Friml et al., 2003a; Friml et al., 2003b). To determine whether the increase of auxin level under high pH conditions occurred due to defective PAT, we next examined the subcellular localization of PIN proteins under alkaline conditions. Notably, we observed an altered subcellular localization of PIN7 at pH 8.0. In particular, PIN7 formed strong intracellular signals in columella cells, which was confirmed by quantitative analysis of the ratio between cytoplasm and plasma membrane (**Figures 3A, D**). Because PIN7 is usually localized on the PM, which is essential for auxin efflux and the maintenance of auxin maximum in root stem cells, the ectopic localization of PIN7 under alkaline stress may change the local auxin gradient (**Figure 2**).

As both PIN3 and PIN7 are distributed in columella cells, we also compared the subcellular localization of PIN3 and PIN7 under alkaline conditions. However, we did not detect significant change of PIN3 membrane localization at pH 8.0 (**Figures 3A, D**). This finding indicates that the change of subcellular localization in columella cells under alkaline stress is unique to PIN7.

### PIN7 was translocated into the vacuole lumen under alkaline stress

Next, to determine the subcellular compartment where PIN7 was translocated, we crossed different organelle marker lines with the *PIN7pro::PIN7–GFP* (hereafter refers to *PIN7-GFP*) transgenic line, and detected the colocalization pattern under alkaline conditions. We found that the PIN7 signal within the cells was circled by the tonoplast marker *mCherry-VAMP711* (Geldner et al., 2009) at pH 8.0 (**Figure 3B**), but showed no obvious colocalization with the TGN/EE marker *HAP13-RFP* (Wang et al., 2013) (**Figure S3**), indicating that PIN7 is transported to the vacuole lumen under alkaline stress.

Interestingly, we also observed that the vacuolar transport of PIN7 was reversible. When 3-day-old seedlings grown on a medium with pH 8.0 were transferred to a medium with pH 5.8, the vacuole lumen distribution of PIN7 disappeared. However, those transferred to a medium with pH 8.0 did not disappear (**Figures 3C, E**). By contrast, when seedlings grown on medium with pH 5.8 were transferred to a medium with pH 8.0, PIN7 was transported to the vacuole lumen as expected (**Figures 3C, E**). These findings strongly suggest that the vacuolar transport of PIN7 is induced by alkaline stress in a reversible manner, which may play a fundamental role in root alkaline response.

### PIN7 is a key player in the root alkaline response

**Figure 4:**
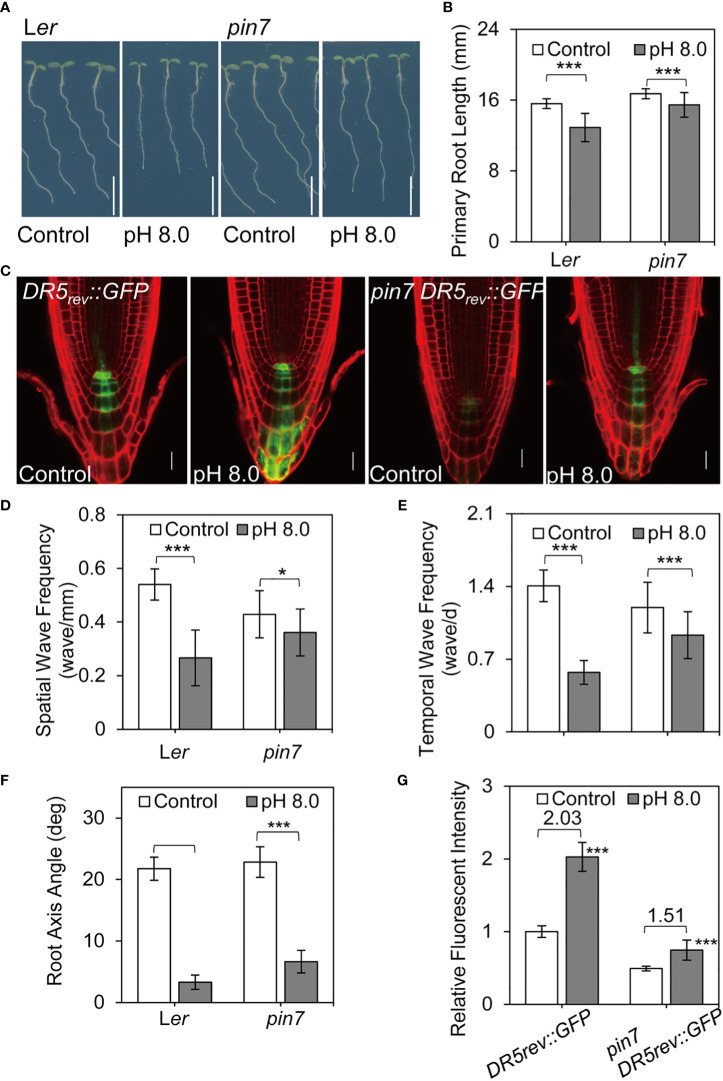
PIN7 plays a crucial role in auxin accumulation and root waving phenotype under alkaline stress. **(A)** The root phenotype of 6-d-old wild-type seedlings and pin7 mutants under control (pH 5.8) or alkaline (pH 8.0) conditions. Scale bar, 5 mm; **(B)** Quantification of the primary root length as demonstrated in **(A)** Values are mean ± SD (n > 40 seedlings). **(C)** Auxin distribution pattern as revealed by the DR5rev::GFP reporter in wildtype seedlings and pin7 mutants under control (pH 5.8) or alkaline (pH 8.0) conditions. **(D, E, F)** Statistical analysis of the spatial wave frequency (wave/mm) **(D)**, temporal wave frequency (waves/d) **(E)**, and root axis angle **(F)** in wild-type seedlings and pin7 mutants as shown in **(A)** (n > 40 seedlings). **(G)** Relative auxin level as revealed by the DR5rev::GFP reporter in wild-type and pin7 mutants as shown in **(C)** (n > 15). Statistically significant differences were determined by using student’s t-test (* P < 0.05; *** P < 0.001).

Next, we examined the root growth phenotypes of *pin7* mutants under alkaline conditions. Interestingly, the *pin7* mutants largely retained the root waving phenotypes under alkaline stress compared with the control plants (**Figures 4A, B** **, D–F**, **Figure S4A**). Furthermore, the change of *DR5rev:GFP* signal between pH 5.8 and pH 8.0 conditions was less obvious in *pin7* mutants than in wild-type plants (**Figure 4C**). We speculated that this is caused by altered auxin level. Quantification of fluorescence intensity indicated that the *DR5rev:GFP* signal at pH 8.0 was 2.03-fold higher than that at pH 5.8 in wild-type seedlings. However, the GFP intensity at pH 8.0 was only 1.51-fold higher than that at pH 5.8 in *pin7* mutant (**Figure 4G**), suggesting that PIN7 is important for the enhanced auxin response under alkaline stress.

Notably, although both PIN3 and PIN7 localize at columella cells, the *pin3-4* (Young et al., 2006) mutants exhibited a similar response to alkaline stress as the control seedlings (**Figure S4B**, **Figures S5A** **–D**). Previous study revealed that PIN2 is transferred to vacuole lumen under dark conditions (Gao et al., 2014). Therefore, we also examined the growth phenotype of *eir1-1* (*pin2* mutants) (Luschnig et al., 1998) under alkaline stress. Although the *eir1-1* mutants showed strong defects in gravitropism as reported before, the root waving phenotype was still significantly reduced under alkaline condition (**Figures S5E** **–H**). This is very different from the *pin7* mutants, which still retained the root waving phenotype at high pH (**Figures 4A–B** **, D–F**).

Altogether, our findings suggest that PIN7 is crucial for auxin accumulation at columella cells in root cap at high pH conditions and therefore is important for proper root morphology under alkaline stress.

PIN7 protein is translocated to the vacuole lumen through vacuolar sorting pathways under alkaline stress

**Figure 5:**
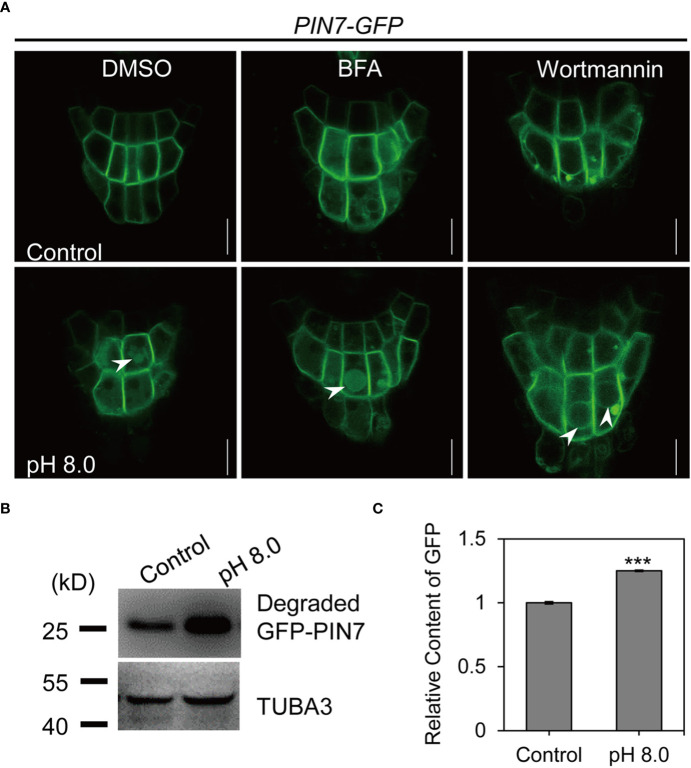
PIN7 protein is transported to vacuole lumen through vacuolar sorting pathways under alkaline stress. **(A)** BFA treatment does not interfere with the vacuole lumen localization of PIN7 under alkaline conditions while wortmannin treatment abolishes this process. PIN7–GFP transgenic plants were grown on 1/2 MS medium at pH 5.8 for 5d and were then transferred to medium at pH 5.8 (control) or pH 8.0 with BFA (50 mM) or wortmannin (33 mM) or DMSO overnight, before imaged by confocal microscope. Scale bar, 10 mm. **(B, C)** Western blot and grayscale analysis of the released free GFP from PIN7–GFP fusion protein under control (pH 5.8) or alkaline (pH 8.0) conditions. Statistically significant differences were determined by using student’s t-test (*** P < 0.001).

In plants, PM-localized proteins are transported to the vacuole through sequential sorting components (Heucken and Ivanov, 2018). We next examined whether the vacuolar transport of PIN7 depends on endosomal trafficking pathways by treatment with trafficking inhibitors. We first transferred 5-day-old seedlings to growth medium of pH 5.8 or pH 8.0 with BFA or DMSO and incubated overnight. BFA is a potent inhibitor of endosomal recycling by targeting the ARF-GEFs and induces intracellular aggregates (so-called BFA bodies) (Geldner et al., 2001; Geldner et al., 2003). We found that BFA treatment (50 μM) did not change the vacuole localization of PIN7 under alkaline stress (**Figure 5A**). By contrast, wortmannin (33 μM), a phosphatidylinositol 3-kinase inhibitor that induces MVB fusion (Wang et al., 2009), completely blocked the vacuolar targeting of PIN7 under the same conditions (**Figure 5A**). We also tested the response to BFA by short-term treatment. 45 min treatment with 50 μM BFA induced PIN7 aggregates colocalized with the FM4-64 labeled BFA bodies at PH 5.8 (**Figure S6**). However, BFA still did not disrupt the vacuole localization of PIN7 at PH 8.0, indicating that the vacuolar transport of PIN7 may not require the function of ARF-GEFs. This is very different from PIN2, whose vacuolar transport under dark conditions is very sensitive to BFA treatment (Kleine-Vehn et al., 2008). The above results indicate that PIN7 might be transported to the vacuole lumen through vacuolar sorting pathway independent of the ARF-GEFs but requires proper function of MVBs.

To confirm that the membrane protein PIN7 was degraded after entering the vacuole lumen, we quantified the released GFP under alkaline stress *via* grayscale analysis by using tubulin as an internal control. Our results showed that the abundance of free GFP released from the PIN7–GFP fusion protein after alkaline treatment was significantly higher than that in control samples (**Figures 5B, C**), indicating that PIN7 is indeed degraded after transferred to the vacuole lumen.

Taken together, these results suggest that PIN7 is transported to vacuole lumen for degradation through vacuolar sorting pathway under alkaline stress.

### The ESCRT component FREE1 is essential for the vacuolar transport of PIN7 and normal root response under alkaline stress

We have shown that the vacuolar transport of PIN7 requires normal function of MVBs. Previous study reported that the ESCRT component endosomal sorting 1 (FREE1) is an important regulator of MVB biogenesis. FREE1 also facilitates the vacuolar transport of PIN2 under dark conditions (Gao et al., 2014). Therefore, we assessed whether the vacuolar transport of PIN7 was mediated *via* a FREE1-dependent sorting pathway by crossing *PIN7–GFP* transgenic line with the FREE1 induced silence line (*DEX-FREE1-RNAi*). Notably, the PIN7 vacuole localization disappeared when FREE1 was induced to be silence (**Figure S7**). This result suggested that the vacuolar targeting of PIN7 requires the proper function of FREE1.

**Figure 6:**
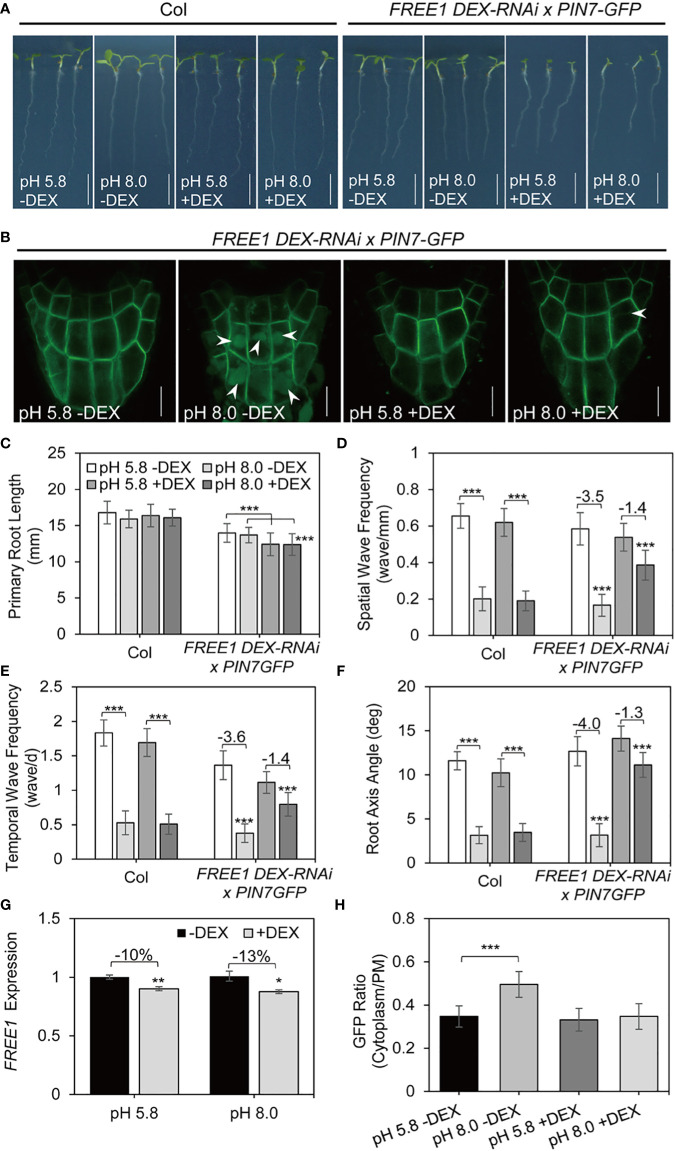
FREE1 is required for PIN7 vacuolar transport and is essential for normal root response under alkaline stress. Data presented in this figure used wild-type Col seedlings or the crossed lines between FREE1 DEX-RNAi and PIN7–GFP. **(A, B)** Seedlings were grown on medium at pH 5.8 (control) or pH 8.0 with or without DEX (10 nM) induction for 6 d. (n > 35). Scale bar, 5 mm. **(A)** Representative growth phenotypes. **(B)** Confocal microscope image of PIN7 in columella cells. **(C)** Quantification of the primary root length in seedlings as demonstrated in (A) Values are mean ± SD (n > 35 seedlings). **(D, E, F)** Statistical analysis of the spatial wave frequency (waves/mm) **(D)**, temporal wave frequency (waves/d) **(E)**, and root axis angle **(F)** in seedlings as demonstrated in (A) Each experiment was repeated three times (n > 35). **(G)** Normalized transcription level of FREE1 under pH 5.8 (control) or pH 8.0 with or without DEX (10 nM) treatment. **(H)** Quantitative analysis of the signal ratio between cytoplasm and plasma membrane (PM) in columella cells of PIN7-GFP. Statistically significant differences were determined by using student’s t-test (* P < 0.05; ** P < 0.01; *** P < 0.001).

We next assessed whether the regulation of PIN7 by FREE1 is important for root response to alkaline stress. We used the same crossed line between *PIN7–GFP* and *DEX-FREE1-RNAi* for phenotype analysis. In the absence of dexamethasone (DEX) induction, the crossed seedlings exhibited similar response to alkaline stress compared with the control seedlings (**Figures 6A** **, C–F**). Because high-level DEX induction results in severe growth defects even seedling death (Gao et al., 2014), we changed to low-level induction instead to ensure that seedlings could be phenotyped (10 nM DEX, **Figure 6G**). Under this condition, the main root length of FREE1-RNAi seedlings was slightly reduced after induction but the cotyledon size was obviously reduced (**Figures 6A, C**). Nonetheless, the root waving phenotype was largely retained in FREE1-RNAi mutants at high pH conditions (**Figures 6A** **, D–F**). In addition, PIN7 failed to be transferred to vacuole lumen under the same condition (**Figures 6B, H**). By contrast, *snx1-2*, the mutant of retromer subunit, showed normal response to alkaline stress similar to control plants (**Figure S8**), indicating that FREE1 specifically participates in the regulation of root waving phenotype under alkaline stress.

In summary, our data suggest that the vacuolar transport of PIN7 depends on proper function of ESCRT component FREE1, which is essential for normal root response under alkaline stress.

## Conclusion

**Figure 7:**
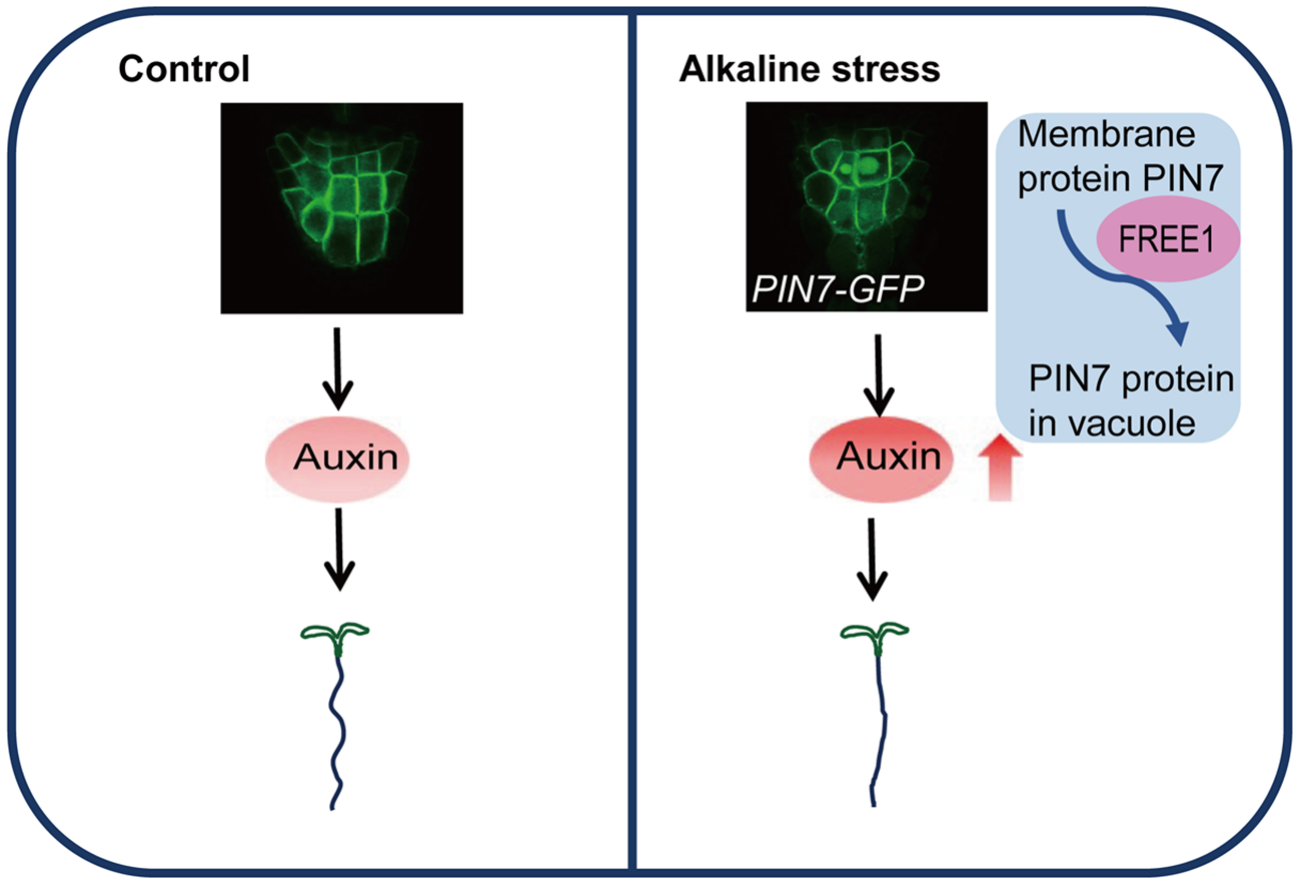
Proposed model of PIN7-regulated root morphology under alkaline stress. A simplified model describes the PIN7-regulated root morphological changes under alkaline stress based on the major conclusions from this work. (1) Under alkaline conditions, auxin accumulation in root columella cells results in reduced root waving pattern. (2) The vacuolar transport of PIN7 is responsible for the increased auxin level and thus normal root response to alkaline stress. (3) The ESCRT component FREE1 is a key factor regulating PIN7 vacuolar transport and proper root morphology under alkaline conditions.

In this work, we report the change of root waving phenotypes at high pH (pH 8.0). We also demonstrate that PIN7 is transported to vacuole lumen under the same alkaline condition. This cellular process depends on the ESCRT subunit FREE1, which is essential for PIN7 vacuolar transport and normal root response to alkaline stress. The major conclusions are summarized as the working model in **Figure 7**.

## Discussion

Alkaline stress impairs the root waving pattern depending on PIN7 localization

Alkaline stress is a major and destructive form of abiotic stress in plants and causes serious crop losses. Intense alkaline stress is known to inhibit the root and meristem length (Li et al., 2015). Our results are consistent with previous findings, which state that the primary root growth is reduced at very high pH conditions (pH 9.0). Under moderate high pH conditions (pH 8.0) when the main root growth is not obviously inhibited, we observed a significant reduction in root waving pattern. We also proved that the reduced root waving pattern under high pH conditions (pH 8.0) is caused by ectopic intracellular localization of PIN7.

Auxin distribution contributes to the plasticity of plant growth and regulates plant adaptation to external environmental changes (Friml, 2003; Lv et al., 2021). For example, the alkaline environment can affect AUX1 and stimulate the expression of auxin biosynthesis-related genes *via* the ethylene pathway, thereby increasing auxin accumulation and inhibiting root elongation (Li et al., 2015). The intracellular reorientation of PIN2 leads to the redistribution of auxin and is involved in root growth, showing geotropism and avoidance of high salt concentrations (Abas et al., 2006; Galvan-Ampudia et al., 2013). In our study, we found that the intracellular localization of PIN7 leads to increased auxin level in root caps. Although both PIN2 and PIN7 can be transferred to vacuole lumen, our data demonstrated that only the *pin7* but not *eir1-1* mutants largely retained root waving phenotypes under alkaline conditions. Both PIN3 and PIN7 localize at columella cells in root cap. However, only PIN7 but not PIN3 was translocated to vacuole lumen at high pH conditions; only *pin7* but not *pin3-4* mutants disrupted the normal root response under alkaline stress. All the data suggest that PIN7 is a unique auxin transporter that specifically responds to alkaline stress and is essential to maintain normal root morphology under alkaline conditions. Our study shed light on the functional differentiation of different auxin transporters under environmental stress.

### PIN7 is transported to vacuole lumen *via* a unique sorting pathway under alkaline stress

In our study, we demonstrate that the vacuolar transport of PIN7 is sensitive to wortmannin but resistant to BFA treatment. This is very different from PIN2. The vacuolar transport of PIN2 under dark condition is very sensitive to BFA treatment (Kleine-Vehn et al., 2008). This indicate that, although both PIN2 and PIN7 can be transported to vacuole lumen, the sorting mechanism is different. We also show that even mild inhibition of FREE1 could completely block the vacuolar transport of PIN7. All the pieces of evidence suggest that PIN7 is transported to vacuole lumen under alkaline stress *via* a unique sorting pathway that requires the proper function of FREE1 but might be independent of the ARF-GEFs.

### Inhibition of root waving under high pH conditions is associated with the root cap

The root cap is located at the apex of the root tip and plays a role in protecting the proximal meristem. The root cap is also the sensing and transducing site for several environmental stimuli (Wang et al., 2014). Studies have shown that boundary cells that are shed from root crown cells play a key role in plant defense and in the regulation of the microbial population in the rhizosphere (Driouich et al., 2013). Indole acetic acid (IAA), which is derived from indole butyric acid (IBA) at the root tip, is a source of local auxin, and the root crown participates in root branching (Xuan et al., 2015; He et al., 2017). This suggests that the root cap plays a role in signal transduction, rather than in stem cell protection. Our data showed that the PM-localized PIN7 in columella cells is translocated to vacuole lumen in response to external alkaline stress. Moreover, the abnormal accumulation of PIN7 affects auxin level in root cap. Similar to the role of PIN2 in dark response, our results indicate that PIN7 is a key player in alkaline stress response. PIN7 may help enhance the stress-sensing capacity of root caps by maintaining proper auxin level.

### Role of vacuole sorting pathway for normal root growth under high pH conditions

Plant vacuoles are essential organelles for plant growth and development. They maintain the osmotic pressure of cells and store nutrients and metabolites. Moreover, vacuole plays an important role in plant responses to the environment (Zhang et al., 2014). Members of the ABC superfamily localize at the tonoplast. ABCC1 and ABCC2 play an important role in the detoxification of heavy metals cadmium (Cd) and arsenic (As) (Lv et al., 2022). In addition, vacuole in different crops respond in different manners. In response to water stress, the vacuole membrane of sorghum maintains an intact structure, whereas that of maize leaves is destroyed. This indicates that maintaining the integrity of the vacuole membrane can help plants cope with adverse growth environments (Giles et al., 1976; Zhang et al., 2014).

Here, our data demonstrate that PIN7 is transferred to vacuole lumen for degradation under alkaline conditions, thus revealing a clear link between the alkaline stress response and vacuolar sorting pathway. PM proteins undergo endocytosis and are transported to vacuole for degradation *via* the ESCRT complex (Murphy et al., 2005; Raiborg and Stenmark, 2009; Teis et al., 2009; Belda-Palazon et al., 2016; Moulinier-Anzola et al., 2020). FREE1 is a unique component of plant ESCRT, which is important for vacuole sorting of ubiquitinated membrane proteins (Gao et al., 2014). In this study, we provide several pieces of evidence to support that FREE1 promotes PIN7 protein vacuolar transport to regulate root morphology under alkaline stress. First, the vacuolar transport of PIN7 under alkaline stress is completely blocked when *FREE1* is mildly inhibited. Second, the induced *DEX-FREE1-RNAi* mutants still retain the root waving phenotype at pH 8.0 whereas the *snx1-2* mutants show similar sensitivity as the control. Our work provides new insights about the cytological function of FREE1 in vacuolar transport and highlights its essential role in alkaline stress response.

Root waving reflects the ability of root system to explore the soil substrates. In addition, it is also strongly associated with appropriate lateral root positioning and formation. Therefore, a better understanding of the mechanism by which the alkaline stress affects root morphology may provide new approach to improve agricultural yields in alkaline soil.

## Data availability statement

The data presented in the study are deposited in the NCBI repository, accession number PRJNA872830.

## Author contributions

YL, XL and RL designed most of the experiments. KP and LL also contributed to the experiment design. YL performed most of the experiments and analyzed the data. CM, DD, YY, WX, and SK also helped with the experiments. YL, XL and RL wrote the manuscript. All authors contributed to the article and approved the submitted version.
