# Supplementary material for: Alkaline stress reduces root waving by regulating PIN7 vacuolar transport
Source: Front Plant Sci. 2022 Dec 13;13:1049144. doi: 10.3389/fpls.2022.1049144 (PMC9792863; doi:10.3389/fpls.2022.1049144)
Supplement: Supplementary file 1 [file Presentation_1.pdf]

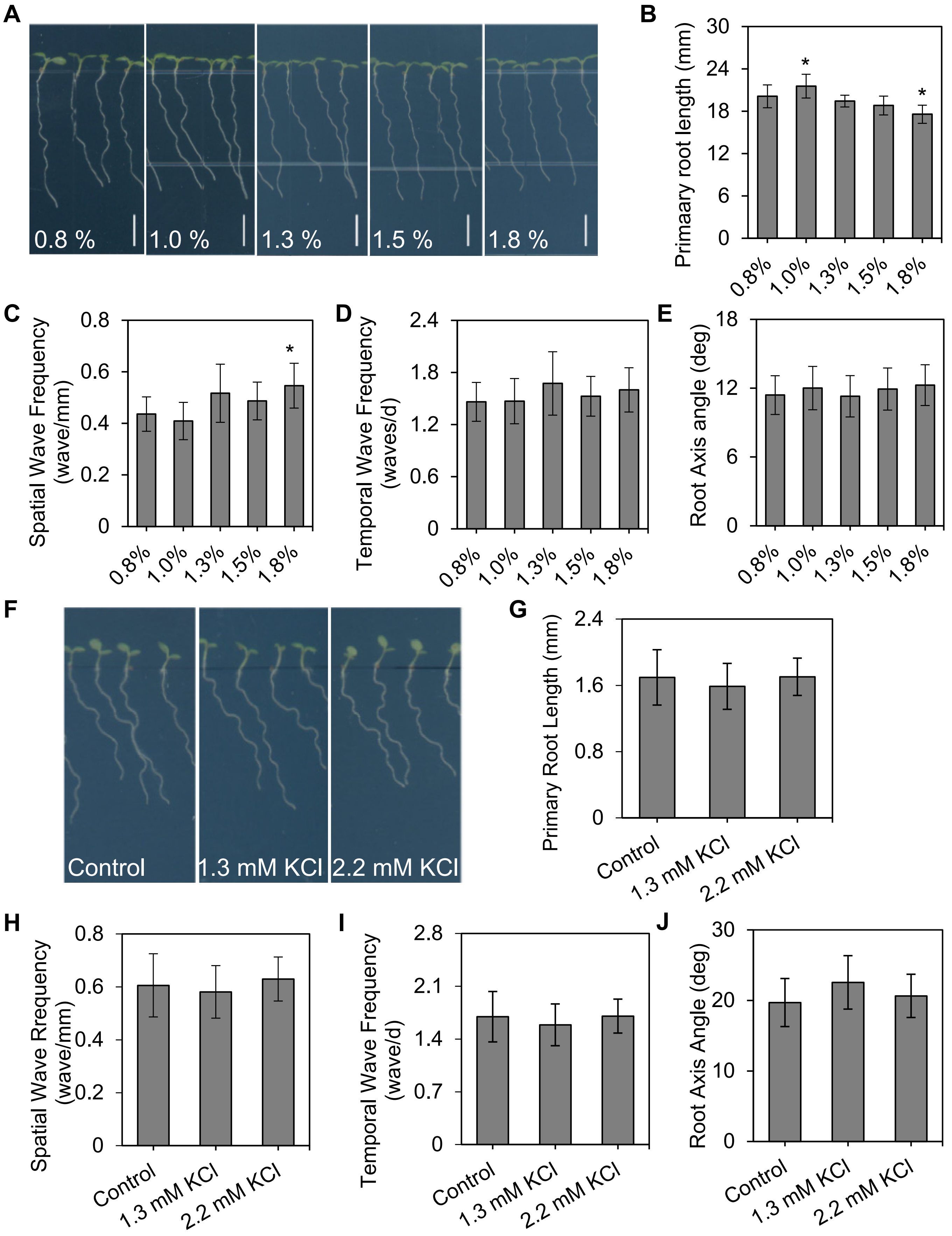

Figure S1. The effects of agar and K^+ concentrations on root waving phenotypes

(A) Root phenotypes of wild-type *Arabidopsis* seedlings on growth medium with different concentrations of agar (0.8%, 1%, 1.3%, 1.5%, or 1.8%) (n > 40). **(B-E)** Statistical analysis of root length **(B)**, spatial wave frequency **(C)**, temporal wave frequency **(D)**, and root axis angle **(E)** as shown in **A** (n > 40). Scale Bar, 5 mm. Each experiment was repeated three times (n > 40). **(F)** Root phenotypes of wild-type *Arabidopsis* seedlings on growth medium without (control) or with 1.3 or 2.2 mM KCl for 6 days. **(G-J)** Statistical analysis of root length **(G)**, spatial wave frequency **(H)**, temporal wave frequency **(I)**, and root axis angle **(J)** as shown in **F** (n > 40). Statistically significant differences were determined by using student's t-test (* $P < 0.05$; ** $P < 0.01$; *** $P < 0.001$).

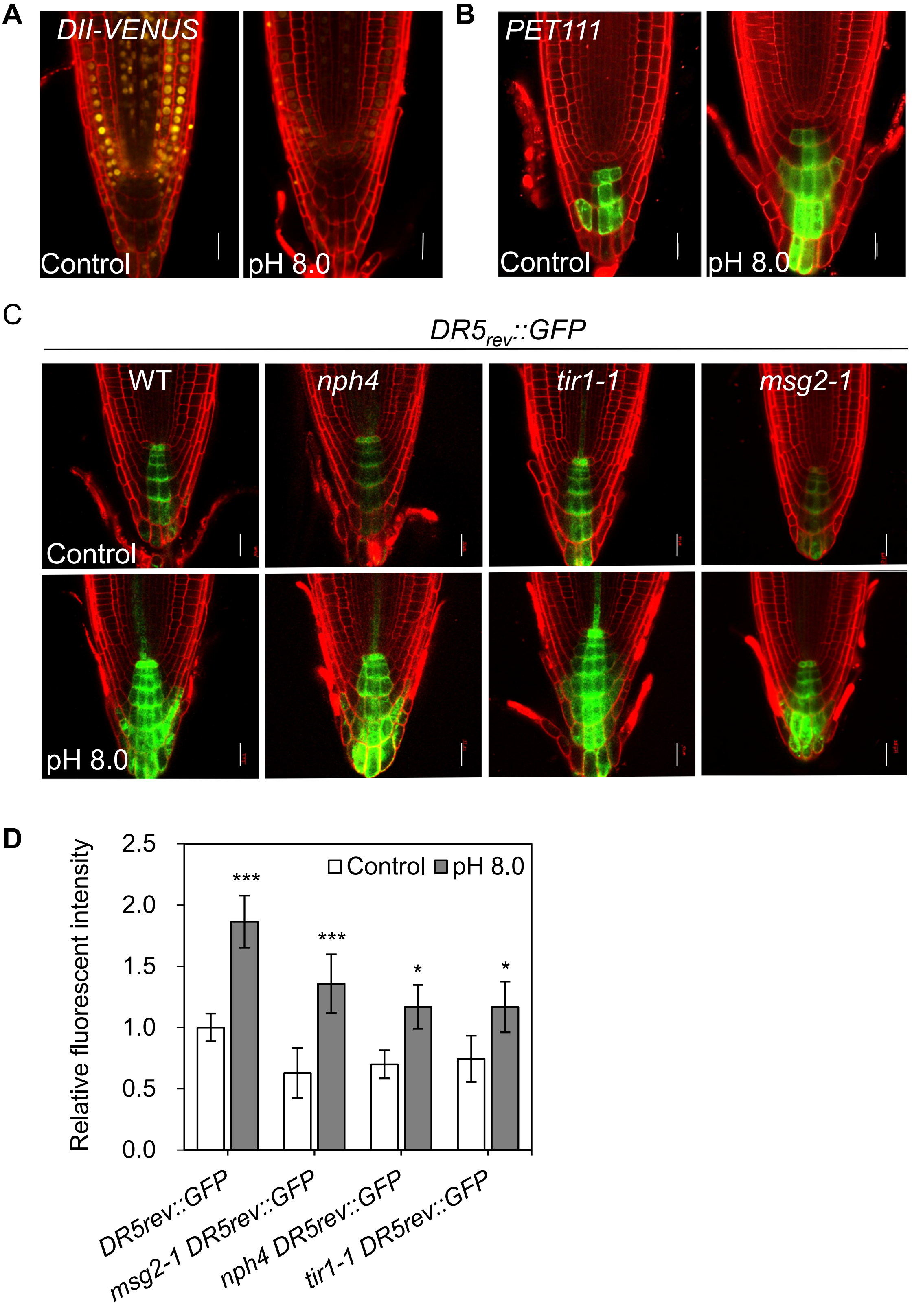

Figure S2. Similar auxin distribution patterns in wild-type and auxin signaling mutants under alkaline stress.

(A) Confocal image of auxin response marker *DII-VENUS* on growth medium at pH 5.8 or pH 8.0 for 6 d. Scale bar, 20 μm. Decreased DII-VENUS signal indicates elevated auxin level. **(B)** Confocal image of root columella cell marker *PET111* on growth medium of pH 5.8 (control) or pH 8.0 for 6 d. Scale bar, 20 μm. **(C)** Confocal image of auxin response marker *DR5rev::GFP* in wild-type seedlings or different auxin signaling mutants on growth medium at pH 5.8 (control) or pH 8.0 for 6 d. The auxin signaling mutants used here are *tir1-1*, *msg2-1*, and *nph4*. **(D)** Statistical analysis of *DR5rev::GFP* reporter as shown in **C.** Scale bar, 20 μm. Statistically significant differences were determined by using student's t-test (* $P < 0.05$; ** $P < 0.01$; *** $P < 0.001$).

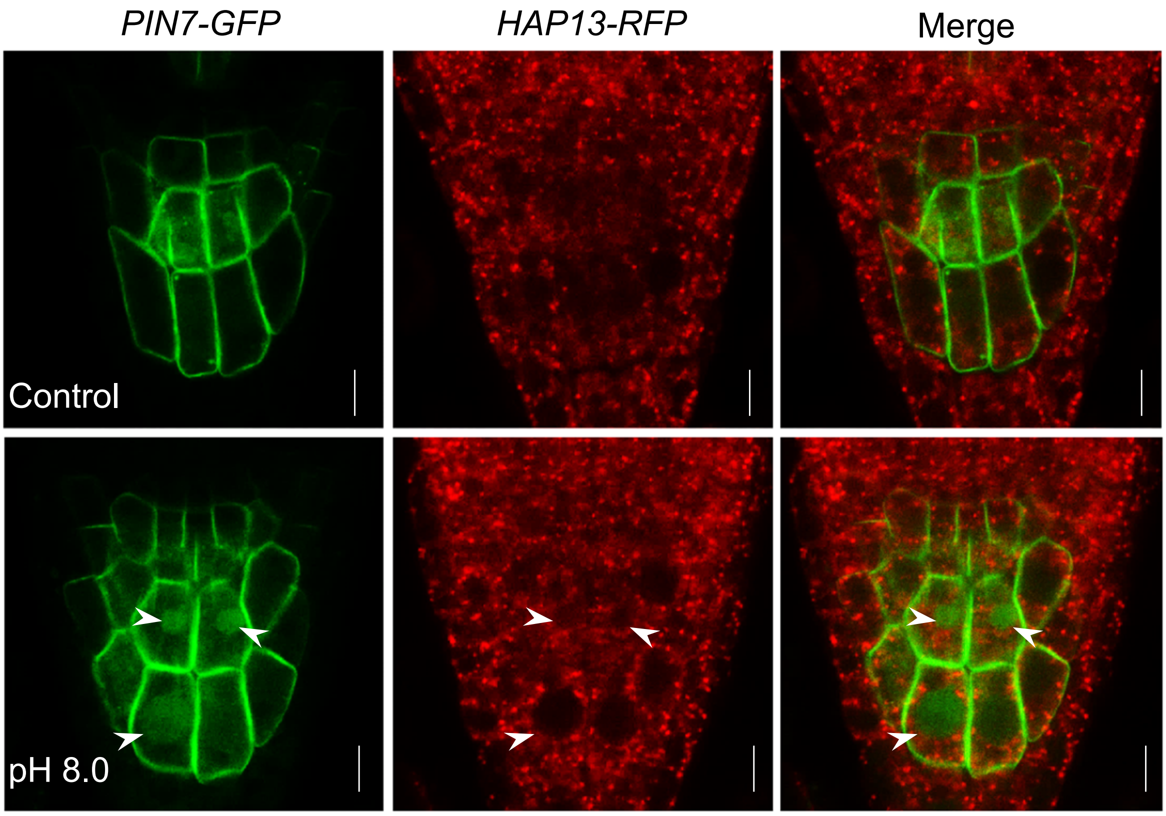

Figure S3. PIN7 does not colocalize with the TGN/EE marker under alkaline stress.

The PIN7–GFP transgenic plants were crossed with the TGN/EE marker *HAP13-RFP*. Under alkaline condition, the intracellular localization of PIN7 does not colocalize with HAP13. Arrows indicate the intracellular distribution of PIN7. Scale bar, 10 μm.

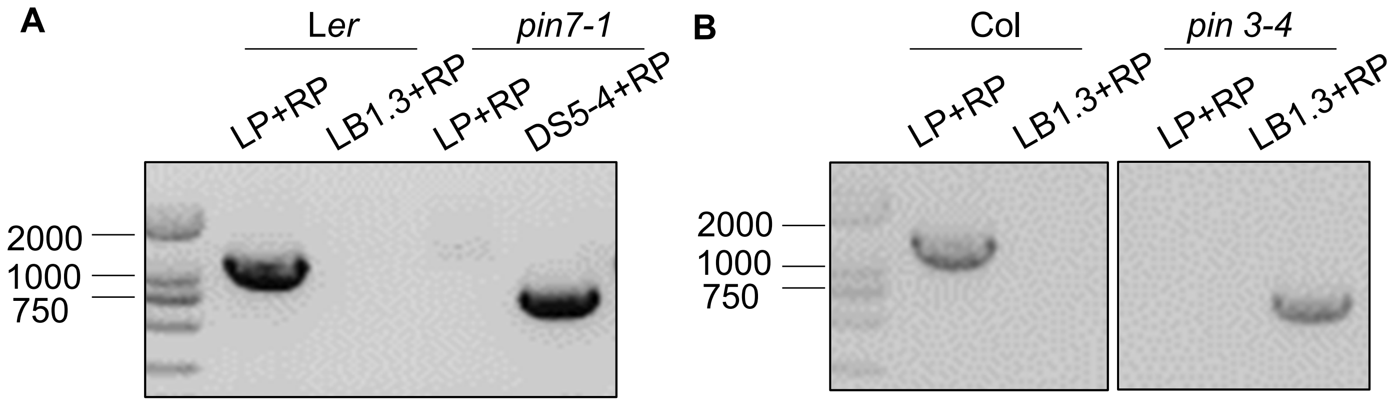

Figure S4. Genotyping results of *pin7-1 and pin3-4* mutants.

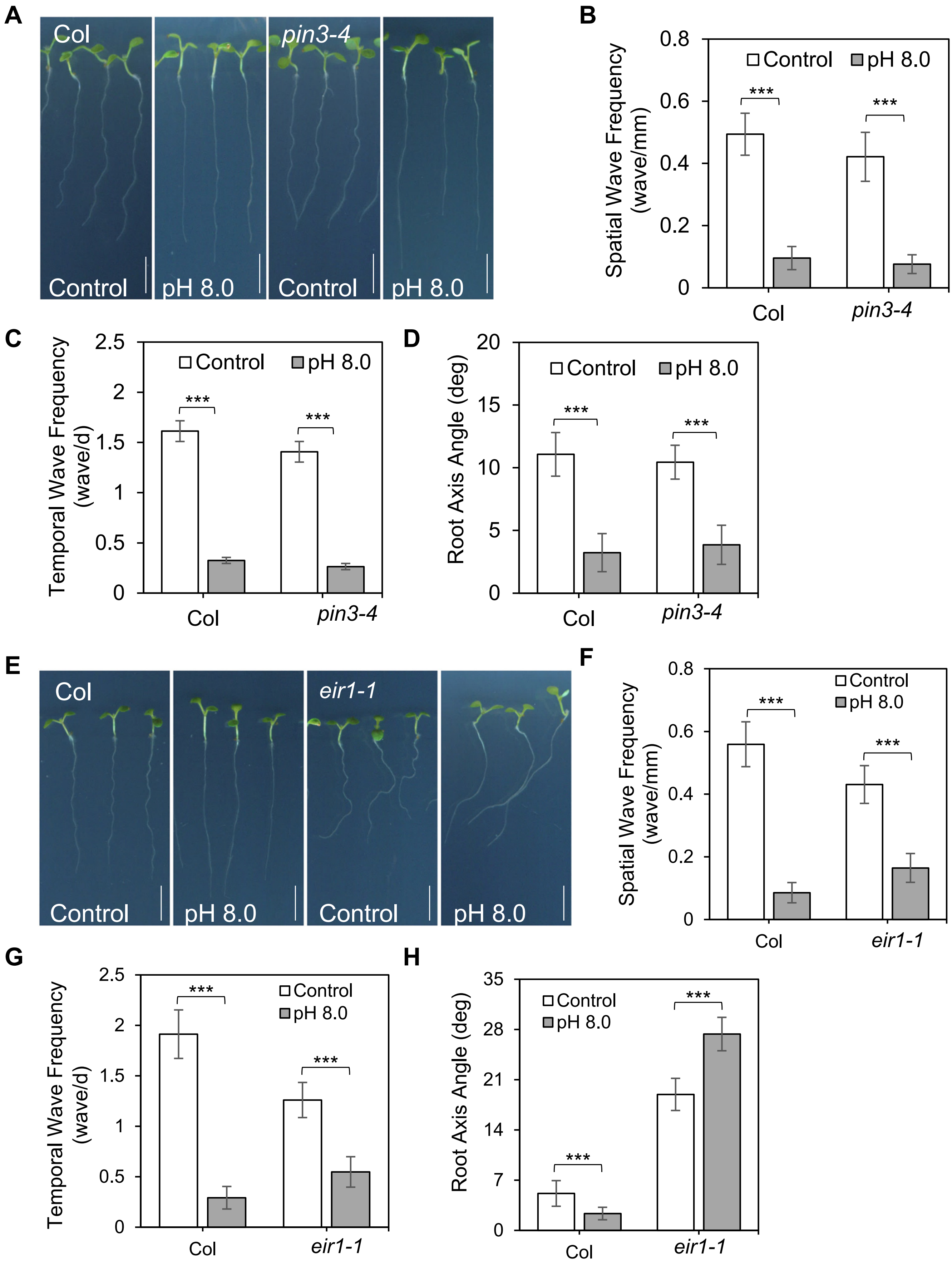

Figure S5. The *pin3* and *pin2* mutants show normal response to alkaline stress.

(A) Root phenotypes of Col and *pin3-4* (Salk_038609) seedlings on growth medium of pH 5.8 (control) or pH 8.0 for 6 d. Scale bar, 5 mm. **(B-D)** Statistical analysis of spatial wave frequency **(B)**, temporal wave frequency **(C)**, and root axis angle **(D)** as shown in **A** (n > 30). **(E)** Root phenotypes of Col and *eir1-1* (*pin2* mutant) seedlings on growth medium of pH 5.8 (control) or pH 8.0 for 6 d. Scale bar, 5 mm. **(F-H)** Statistical analysis of spatial wave frequency **(F)**, temporal wave frequency **(G)**, and root axis angle **(H)** as shown in **E** (n > 30). Statistically significant differences were determined by using student's t-test (* P < 0.05; ** P < 0.01; *** P < 0.001).

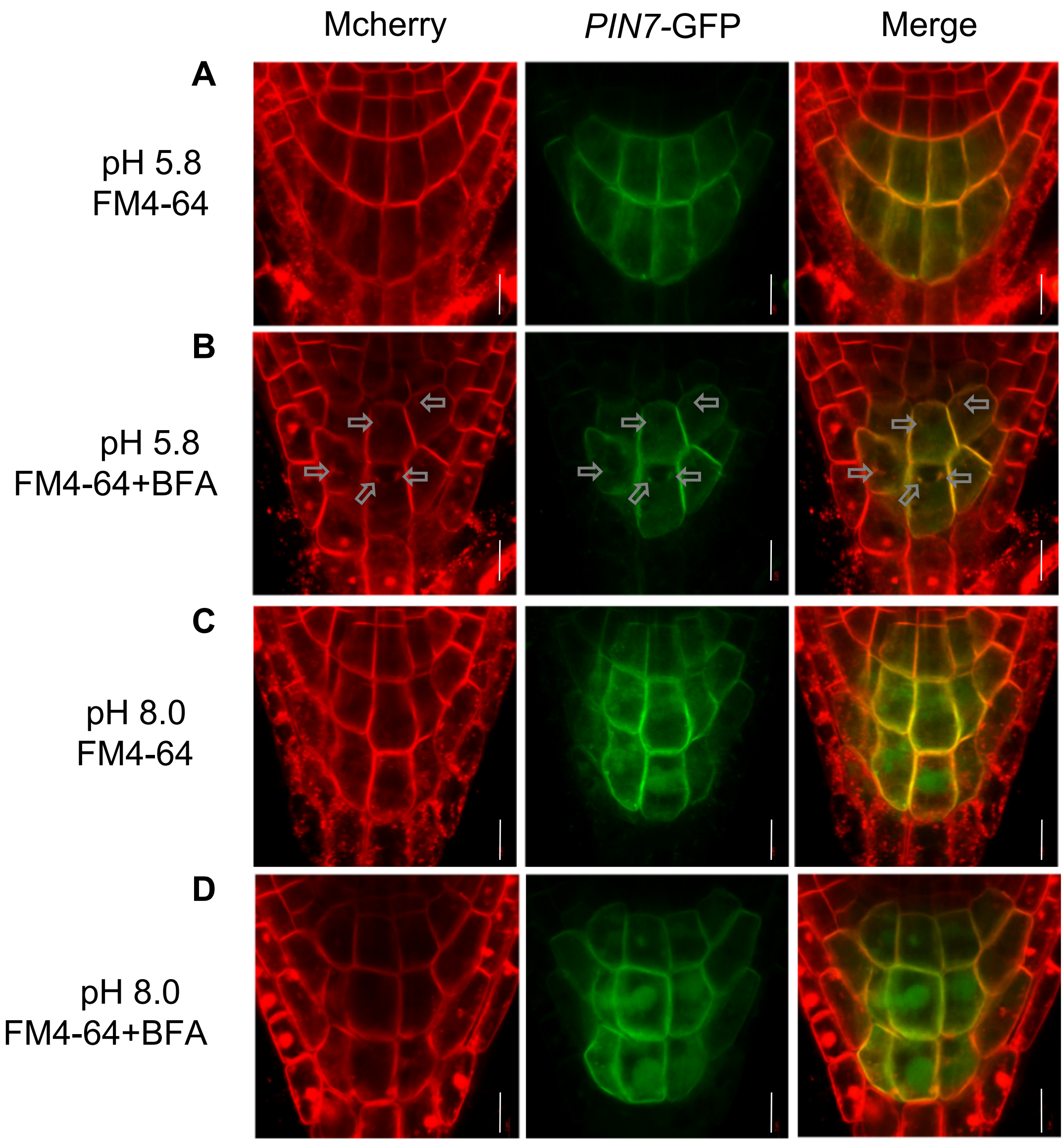

Figure S6. Short-term treatment with BFA does not disrupt the vacuolar transport of PIN7.

The transgenic plant *PIN7-GFP* were grown on 1/2 MS solid medium at pH 5.8 or pH 8.0 for 6 d before treatment. **(A, C)** *PIN7-GFP* seedlings were treated with 1/2 MS liquid medium containing 2 µM FM4-64 for 10 min at pH 5.8 **(A)** or pH 8.0 **(C). (B, D)** *PIN7-GFP* seedlings were pretreated with 1/2 MS liquid medium containing 2 µM FM4-64 for 10 min and then co-treated with 2 µM FM4-64 and 50 µM BFA for 45 min at pH 5.8 **(B)** or pH 8.0 **(D)**. Arrows indicate that PIN7 forms intracellular aggregates colocalized with the FM4-64-labeled BFA bodies. Scale bar, 10µm.

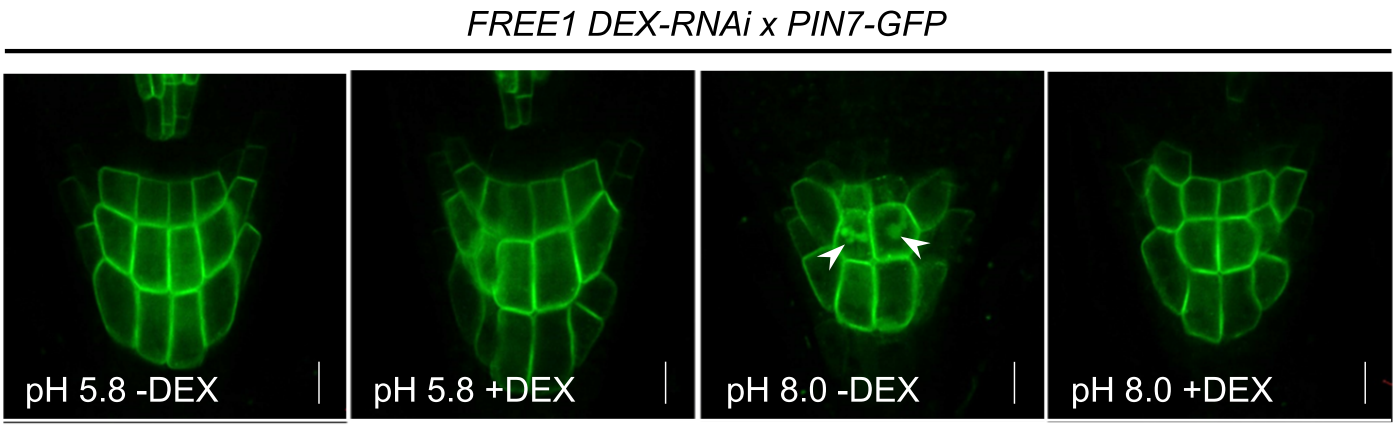

Figure S7. FREE1 is required for PIN7 vacuolar transport

The crossed line between *FREE1 DEX-RNAi* and *PIN7–GFP* were grown on 1/2 MS medium at pH 5.8 for 5 d and were then transferred to medium of pH 5.8 or pH 8.0 with or without DEX (5 µM) for 1 d. Arrows point to the vacuole lumen localization of PIN7. Scale bar, 10 µm.

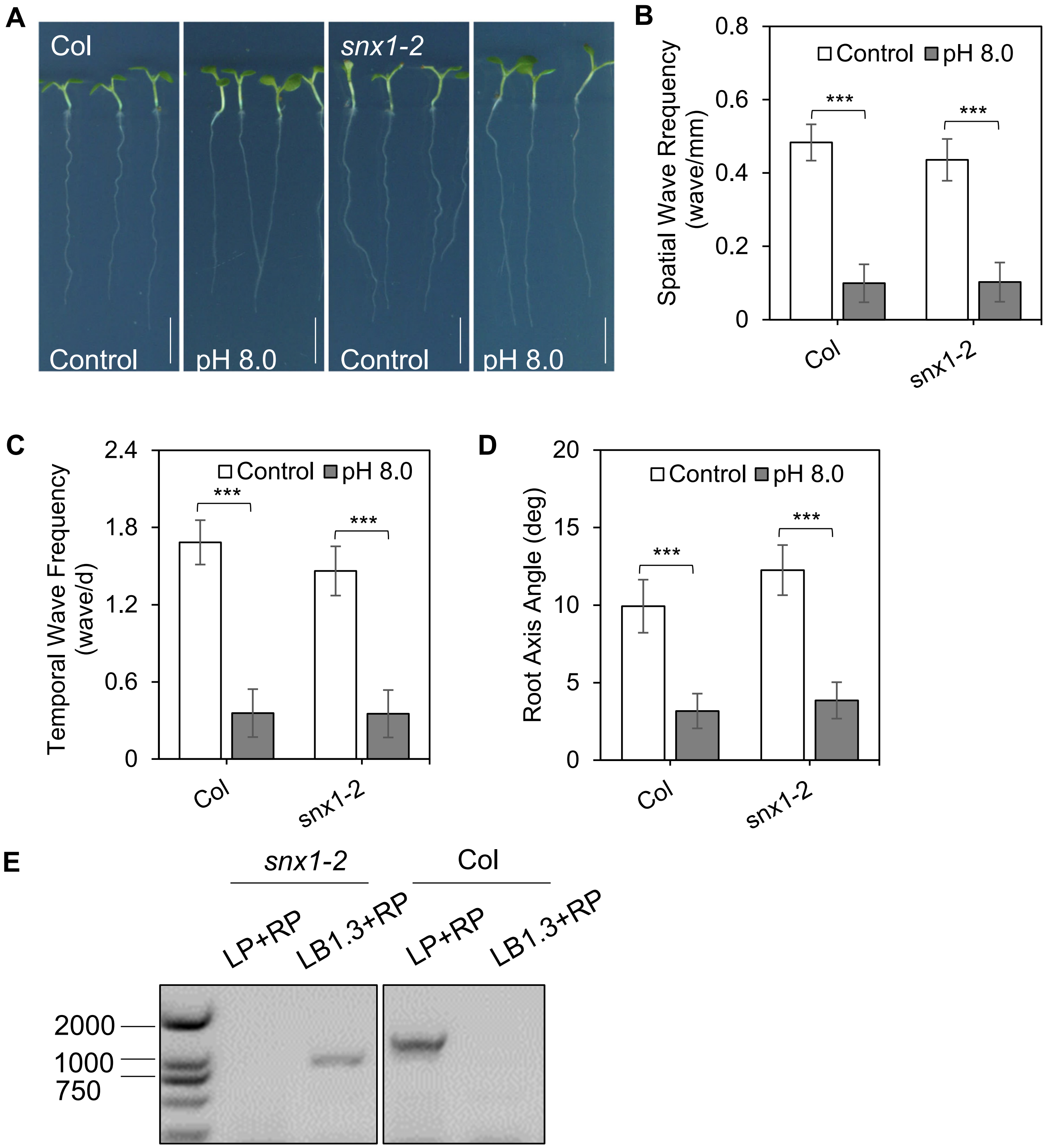

Figure S8. SNX1 is not responsible for the root waving phenotype under alkaline stress.

(A) Root phenotypes of wild-type and *snx1-2* mutants on growth medium of pH 5.8 (control) or pH 8.0 for 6 days (n > 30). Scale bar, 5 mm. **(B, C, D)** Statistical analyses of the spatial wave frequency **(B)**, temporal wave frequency **(C)**, and root axis angle **(D)** as shown in **A**. Each experiment was repeated three times (n > 30). Statistically significant differences were determined by using student's t-test (* P < 0.05; ** P < 0.01; *** P < 0.001). **(E)** Genotyping results of *snx1-2* mutants.

Name	Primer name	Primer sequence (from 5’ start to 3’ end)
pin7-1 (GT4114)	Forward (LP)	CCACTCAAAACAACAAGTGCTG
	DS5-4 Forward	TACGATAACGGTCGGTACGG
	Reverse (RP)	CCGAGCTGGTCTTCAAGTTG
pin3-4 (Salk_038609)	Forward (LP)	TGCCACCTTCAATTCAAAAAC
	Reverse (RP)	TGATTTTCTTGAGACCGATGC
	LB1.3	ATTTTGCCGATTTCGGAAC
snx1-2 (Salk_033351)	Forward (LP)	TCAAGCACCCAAAAGCATTAC
	Reverse (RP)	TGGACAGATTCAGGTTTCAGG

Table S1. Primers used for genotyping.
